# Long-Term Gene Therapy with Thrombospondin 2 Inhibits TGF-β Activation, Inflammation and Angiogenesis in Chronic Allograft Nephropathy

**DOI:** 10.1371/journal.pone.0083846

**Published:** 2013-12-23

**Authors:** Christoph Daniel, Regina Vogelbacher, Andrea Stief, Christina Grigo, Christian Hugo

**Affiliations:** 1 Department of Nephrology and Hypertension, University of Erlangen-Nuremberg, Erlangen, Germany; 2 Department of Pathology, Nephropathology, University of Erlangen-Nuremberg, Erlangen, Germany; 3 Division of Nephrology, Medical Clinic III, University of Dresden, Germany; INSERM, France

## Abstract

We recently identified Thrombospondin-2 (TSP-2) as a regulator of matrix remodelling and inflammation in experimental kidney disease by using TSP-2 null mice and successfully proved TSP-2 overexpression as a therapeutic concept in a short term glomerulonephritis model in the rat. In this current study, we investigated if long-term TSP-2 overexpression is also capable to ameliorate the progression of chronic kidney disease in the setting of the chronic allograft nephropathy F344-Lewis model in the rat. Two weeks after renal transplantation, two rat thigh muscles were transfected once only with either a TSP-2 overexpressing plasmid (n = 8) or a luciferase-expressing plasmid as control (n = 8). Rats were monitored for renal function, histological changes and gene expression in the graft for up to 30 weeks after transplantation. Unexpectedly, only in the TSP-2 treated group 2 rats died before the end of the experiment and renal function tended to be worsened in the TSP-2 group compared to the luciferase-treated controls. In addition, glomerular sclerosis and tubular interstitial injury as well as cortical fibronectin deposition was significantly increased in the TSP-2 treated kidneys despite reduced TGF-β activation and marked anti-inflammatory (macrophages, T-cells and B-cells) effects in this group. Long-term TSP-2 therapy impaired repair of renal endothelium, as demonstrated by significant higher glomerular and peritubular endothelial rarefaction and reduced endothelial cell proliferation in the transplanted kidneys from TSP-2 treated rats compared to controls. This TSP-2 effect was associated with decreased levels of renal VEGF but not VEGF1 receptor. In conclusion, despite its anti-inflammatory and TGF-β activation blocking effects, TSP-2 gene therapy did not ameliorate but rather worsened experimental chronic allograft nephropathy most likely via its anti-angiogenic properties on the renal microvasculature.

## Introduction

Kidney transplantation is the best therapy available for most patients suffering from end-stage renal disease. The mean graft half-life for deceased donor transplants was only 8.8 years in 2005 [1]. Despite improvements in the last 20 years regarding short term graft survival and acute rejection rates [2], long-term graft loss could not be markedly improved [3] [1]. In the meantime several causes for long-term allograft loss have been identified, but no specific treatment options are available. Beside glomerular disease, interstitial fibrosis with tubular atrophy (IF/TA) is one of the most frequent reasons for long-term kidney allograft loss [4]. Like other chronic kidney diseases, chronic allograft nephropathy (CAN) is also predominantly mediated by the key fibrogenic cytokine TGF-β [5] resulting from processes mediated by immunologic and nonimmunologic events as mimicked by the well-established F344-Lewis model in the rat [6]. Repeated measurements of active TGF-β1 plasma levels in renal allograft recipients identified TGF-β1 as an independent predictor for the development of CAN [7]. Microarray analysis using biopsies from transplanted kidneys revealed that genes involved in TGF-β signaling were associated with graft failure [8]. Furthermore, in a rat model for accelerated kidney fibrosis, inhibition of TGF-β expression significantly inhibited renal allograft fibrosis [9].

TGF-β is secreted as an inactive pro-cytokine complex that consists of the mature, active TGF-β protein non-covalently bound to a dimer of its N-terminal propeptide, the so-called latency-associated protein (LAP), and variably to a latent TGF-β binding protein (LTBP). Before it can bind to its receptors, TGF-β has to be activated extracellularly [10]. For different renal diseases like experimental glomerulonephritis and diabetic nephropathy in rodents, we could show that thrombospondin-1 TSP-1 is the major activator of TGF-β [11]. This complex interaction leads to a conformational change within the LAP that allows the mature TGF-β protein to bind to its receptors [12], [13].

Thrombospondin-2 (TSP-2) is the second member of the thrombospondin family, which can also bind the TGF-β procytokine complex, but lacks the ability of its activation [14]. In a recent study in an acute glomerulonephritis model in the rat we used TSP-2 gene therapy in the thigh muscle to competitively block TSP-1 mediated TGF-β activation. Hereby, TSP-2 overexpression resulted in significant reduction of TGF-β activation and subsequent matrix accumulation and inhibited the glomerular proliferative and inflammatory response [15]. In addition, hearts of TSP-2 deficient mice show either age-related or after viral infection an increased fibrotic and inflammatory response [16], [17]. Due to rapid silencing, gene therapy using plasmids with viral promoters is only appropriate for short term therapy over one to two weeks [18]. For the current study the TSP-2 sequence was therefore cloned into a plasmid vector utilizing the eukaryotic ubiquitin promoter resulting in long-term overexpression due to the lack of gene silencing as demonstrated by Gill and coworkers [18].

Therefore, for this study we hypothesized that therapeutical systemic overexpression of TSP-2 is able to positively influence experimental CAN via several different mechanisms such as its anti-inflammatory properties as well as by antifibrotic effects via competition with TSP-1 mediated TGF-β activation. Despite demonstration of anti-inflammatory and TGF-β activation blocking effects, TSP-2 gene therapy did not ameliorate but rather worsened experimental chronic allograft nephropathy most likely via its anti-angiogenic properties on the renal microvasculature.

## Methods

### Animal model and experimental design

The experimental protocol was approved by the german regional committee for animal care and use, which is equivalent to the US IACUC, and authorized by the governmental department (“Regierung von Mittelfranken” Permit number: 54-2532.1-31/08) prior the animal studies were performed in strict accordance with the German welfare act (TierSchG). At the end of the experiment rats were sacrificed by bleeding under isofluran anesthesia. Male F344 (donor) and LEW (recipient) rats (Charles River, Germany) weighting 200 to 250 g were fed standard rat chow (Altromin 1324, Spezialfutterwerke GmbH, Germany) and tap water ad libitum. In donor rats, the left kidney was gently exposed and the renal vein was cut proximal to the vena cava before the kidney was washed with and preserved in ice-cold University of Wisconsin solution. The renal artery was excised with an aortic patch. The ureter was cut next to the bladder. Kidney was grafted heterotopically with end-to-side anastomosis to the recipient's aorta and vena cava, respectively. The ureter was anastomosed end-to-end with individual stitches. Cold ischemia was approximately 50 min; warm ischemia time was 35 min on average. The native left kidney was removed during transplantation; the remaining right kidney was removed 10 days later. During the first 10 days after transplantation, rats received 5 mg cyclosporine per kg body weight by gavage to prevent acute rejection. Two weeks after transplantation, the animals were divided into two groups and treated either with the luciferase expressing plasmid (pUblux) as placebo (P; n = 8), or with a TSP-2 expressing plasmid (pUbTSP-2; n = 8) using dual muscle transfection. Two animals were sacrificed during the study due to rapid loss of weight. Survival analysis was tested using the Kaplan-Meier log-rank test, and showed no significant difference between the two groups. A serum sample and a 24-h urine collection for measuring proteinuria, serum creatinine and urea were performed in periodic intervals. The rats were sacrificed 30 weeks after transplantation.

### Gene therapy

For gene therapy we used the pUbLux vector for long-term gene expression (kindly provided by D.R. Gill and S.C. Hyde, Department of Clinical Laboratory Sciences, University of Oxford, UK). The luciferase gene (Lux) was replaced by a multiple cloning site (MCS). Briefly, *mTSP-2* was cut from pcDNA3.1 (kindly provided by M. Streit, Department of Surgery, Massachusetts General Hospital and Harvard Medical School, Boston, USA) using *Eco*RI and XbaI and subsequently ligated into pUbMCS digested equally. Transfection of left and right thigh muscles with 35 µg plasmid DNA containing mouse TSP-2 or luciferase DNA (control) was performed as descibed previously [15]. For evaluation of long-term expression, the ubiquitin-driven luciferase plasmid was compared with the commercially available pGl2 vector using the transfection conditions as mentioned above. Seven days, 4 months and one year after transfection, the luciferase activity was evaluated in vivo by intravenous injection of 1 mg luciferin dissolved in 1 ml PBS and recording over a 300-second integration period by a cooled CCD camera system (Hamamatsu C4742– 98; Hamamatsu Photonics, Okayama City, Japan) immediately after injection of luciferin.

### Renal morphology and immunohistochemistry

Renal biopsies were fixed in methyl Carnoy's solution, embedded in paraffin, and cut into sections of 3 µm- for indirect immunoperoxidase staining as described elsewhere [19]. Sections were also stained with the periodic acid Schiff reagent (PAS) and counterstained with hematoxylin. For each biopsy, 40 cortical glomerular cross-sections were evaluated in a blinded fashion by two independent observers.

Glomerulosclerosis was determined using PAS stained sections as described [20]. Glomerular hypertrophy was determined by measuring the glomerular tuft area of 50 glomerular cross-sections with computer-assisted morphometry using Metavue software (Visitron GmbH, Puchheim, Germany). All immunohistological evaluations were performed in a blinded fashion by two independent observers.

The following antibodies were used in this study: A murine IgM monoclonal antibody (mAb) against the proliferating cell nuclear antigen (PCNA) (PC10, DAKO, Glostrup, Denmark); ED-1, a murine IgG_1_ mAb to a cytoplasmic antigen present in monocytes, macrophages and dendritic cells (Serotec, Ltd., Oxford, United Kingdom [21]); MCP-1, a rabbit polyclonal Ab recognizing macrophage chemoattractant protein-1 (Santa Cruz Biotechnology Inc., Santa Cruz, CA); CD8a, a murine IgG1 mAb specific for cytotoxic T-cells (BD biosciences, Heidelberg, Germany); CD45R, a murine IgG1 mAb specific for B-cells (BD biosciences); MHC class II, a murine IgG1 mAb specific antigen presenting cells (Serotec); CD31, a murine IgG mAb specific for rat PECAM1 on endothelial cells [22]; TSP-2, a polyclonal goat antibody raised against recombinant human TSP-2 (R&D systems, Wiesbaden-Nordenstadt, Germany); TSP-1, a murine IgG_1_ mAb, clone A6.1 specific for TSP-1 (Labvision, Fremont, CA, USA [23]). Myofibroblasts were stained with a murine IgG_2_ mAb recognizing α-smooth muscle actin (DAKO, Hamburg, Germany [23]). Immunostaining for matrix proteins was conducted with polyclonal antibodies to collagen IV (goat anti-human/bovine collagen IV, Southern Biotechnology Associates, Inc., Birmingham, AL [24]); fibronectin (rabbit anti-rat fibronectin, Labvision). The TGF-β system was studied using antibodies to TGF-β1 (rabbit anti-human TGF-β1, Santa Cruz Biotechnology Inc. [23]); TGF-β2 (rabbit anti-human TGF-β2, Santa Cruz [23]); active TGF-β1 (chicken anti-human active TGF-β1, (R&D systems, Germany [23], [25], Phospho-Smad2/3 (rabbit anti-human Smad2 peptide phosphorylated at Ser-433/435, Santa Cruz [23]) or PAI-1, a rabbit polyclonal to human plasminogen activator inhibitor-1 (Santa Cruz). Furthermore, a polyclonal goat anti-rat VEGF antibody (R&D systems) and a goat anti-rat VEGFR1 antibody (Labvision) and a murine monoclonal antibody for human desmin (DAKO) were used.

Negative controls for immunostaining included either deletion or substitution of the primary antibody with equivalent concentrations of an irrelevant murine monoclonal antibody or preimmune rabbit IgG. After incubation with primary antibodies overnight at 4°C, specific biotinylated secondary antibodies (all by Vector Lab., Burlingame, CA, USA) were applied to tissue sections, followed by peroxidase-conjugated Avidin D (Vector), and colour development with diaminobenzidine without nickel chloride, and histogreen (Linaris GmbH, Wertheim-Bettingen, Germany) for nuclear staining.

Glomerular expression of collagen IV, fibronectin, total TGF-β1, total TGF-β2, active TGF-β, TSP-1, TSP-2, VEGF and PAI-1 was graded semiquantitatively as described previously [26] and reflected changes in the area and intensity of the glomerular tuft. In addition, the average number of CD8a, MHC class II, CD45R, ED-1 or PCNA positive cells per glomerular cross-section was determined.

### Real-time quantitative RT-PCR

For the evaluation of renal mRNA levels in the kidney transplantation model, RNA was purified from isolated glomeruli collected at the end of the experiment. Glomeruli were lysed in RLT-buffer and RNA was purified using Rneasy micro-columns (both from Qiagen, Hilden, Germany) with subsequent DNAse digestion following manufacturer's instructions. Reverse transcription was performed using reverse transcription reagents from ABgene, Hamburg, Germany following manufacturer's instructions.

Real-time RT-PCR was performed on a TaqMan® ABI 7100 Sequence detection system using the Mastermix (all ABgene, Hamburg, Germany). After an initial hold of 15 minutes 95°C samples were cycled 40 times at 95°C for 15 seconds and 60°C for 60 seconds. The cDNA content of each sample was compared with 18s as housekeeping gene following the ΔΔCt technique [27]. All genes were quantified using the SYBR-GREEN method. Used primer sequences were shown in table 1.

**Table 1:** Primer sequences used for quantification of gene expression by real-time PCR.

Gene	forward primer	reverse primer
18s	5′-TTGATTAAGTCCCTGCCCTTTGT-3′	5′-CGATCCGAGGGCCTCACTA-3′
fibronectin	5′-TTGCAACCCACCGTGGAGTATGTG-3′	5′-CTCGGTAGCCAGTGAGCTTAACAC-3′
SMA	5′-CGGGCTTTGCTGGTGATG-3′	5′-CCCACGATGGATGGGAAA-3′
CTGF	5′-TGTGCACTGCCAAAGATGGT-3′	5′-GGTACACGGACCCACCGA-3′
CD31	5′-CTTCACCATCCAGAAGGAAGAGAC-3′	5′-CACTGGTATTCCATGTCTCTGGTG-3′

### In vitro Proliferation Assay

A rat immortalized glomerular endothelial cell line (RGE cell line [28] was kindly provided by H. Holthöfer, University of Helsinki, Finland) was grown in RPMI-1640 containing 10% fetal calf serum (FCS), 2 mM glutamine and penicillin/streptomycin at 37°C and 5% CO_2_. Proliferation of RGEs treated with 0.05 – 25 µg murine TSP-2 (kindly provided by P. Bornstein, University of Washington, Seattle WA, USA) was monitored by Bromodeoxyuridine (BrdU) uptake during S-Phase using a cell proliferation ELISA (colorimetric) purchased from Roche Diagnostics GmbH, Mannheim, Germany following the manufacturer's instructions. 2,500 rat RGEs were seeded per well of a microtiter plate and starved for 3 days in RPMI-1640 culture medium supplemented with 1% FCS. Pulsing with BrdU was conducted for 4 hours one day after stimulation with 10% FCS. Mean proliferative cell activity from 2 independent experiments performed in triplicate was shown by normalizing the positive control to 100%. Toxic TSP-2 effects could be excluded by measurement of lactat dehydrogenase activity was comparable in supernatants from FCS-stimulated RGE cells treated with 25 µg TSP-2 or without TSP-2 using LDH cytotoxicity detection kit purchased from Takara Bio Inc. (Shiga, Japan) following the manufacturer's instructions.

### Zymography

For zymography, 15 µl serum samples were mixed with loading buffer (1%(w/v) SDS, 1%(w/v) bromphenole blue in 70% glycerol. Serum samples were separated by 10% SDS-PAGE with 0.2% (w/v) gelatine in the resolving gel at 4°C. SDS was removed by agitation of gels for 120 min in 1% (w/v) Triton X 100 in 100 mM Tris, pH 7.5, followed by three subsequent washings for 15 min in activation buffer (100 mM Tris pH 7.5; 10 mM CaCl_2_; 150 mM NaCl; 2 µM ZnSO_4_). After incubation in the same buffer for 20 h at 37°C, gels were stained with 0.1%(w/v) Coomassie brilliant blue R250 in methanol: acidic acid: H_2_O (40∶10∶50) and agitated in the same solution until the stacking gel was destained. Evaluation of protease activity was done by measurement of light intensity of MMP-2 bands using Image J.

### Miscellaneous measurements

Urinary protein was measured using the BioRad Protein Assay (München, Germany) and BSA (Sigma, Deisenhofen, Germany) as a standard. Creatinine and urea in serum or urine were measured using an autoanalyzer (Beckman Instruments GmbH, München, Germany).

### Statistical analysis

Most values are presented as box plots showing the 25–75 percentile within the box and minimum and maximum values as whiskers. Only Luciferase activity, kidney function parameters and *in vitro* endothelial cell proliferation are shown as mean ± SEM. After performing the D'Agostino & Pearson omnibus normality test, statistical significance (defined as p<0.05) was evaluated using the Mann-Whitney U rank test for comparison of control versus TSP-2 group and Anova analysis with Bonferroni posthoc test for endothelial cell proliferation.

## Results

### Gene therapy using eukaryotic ubiquitin-promoter resulted in long-term gene expression

**Figure 1:**
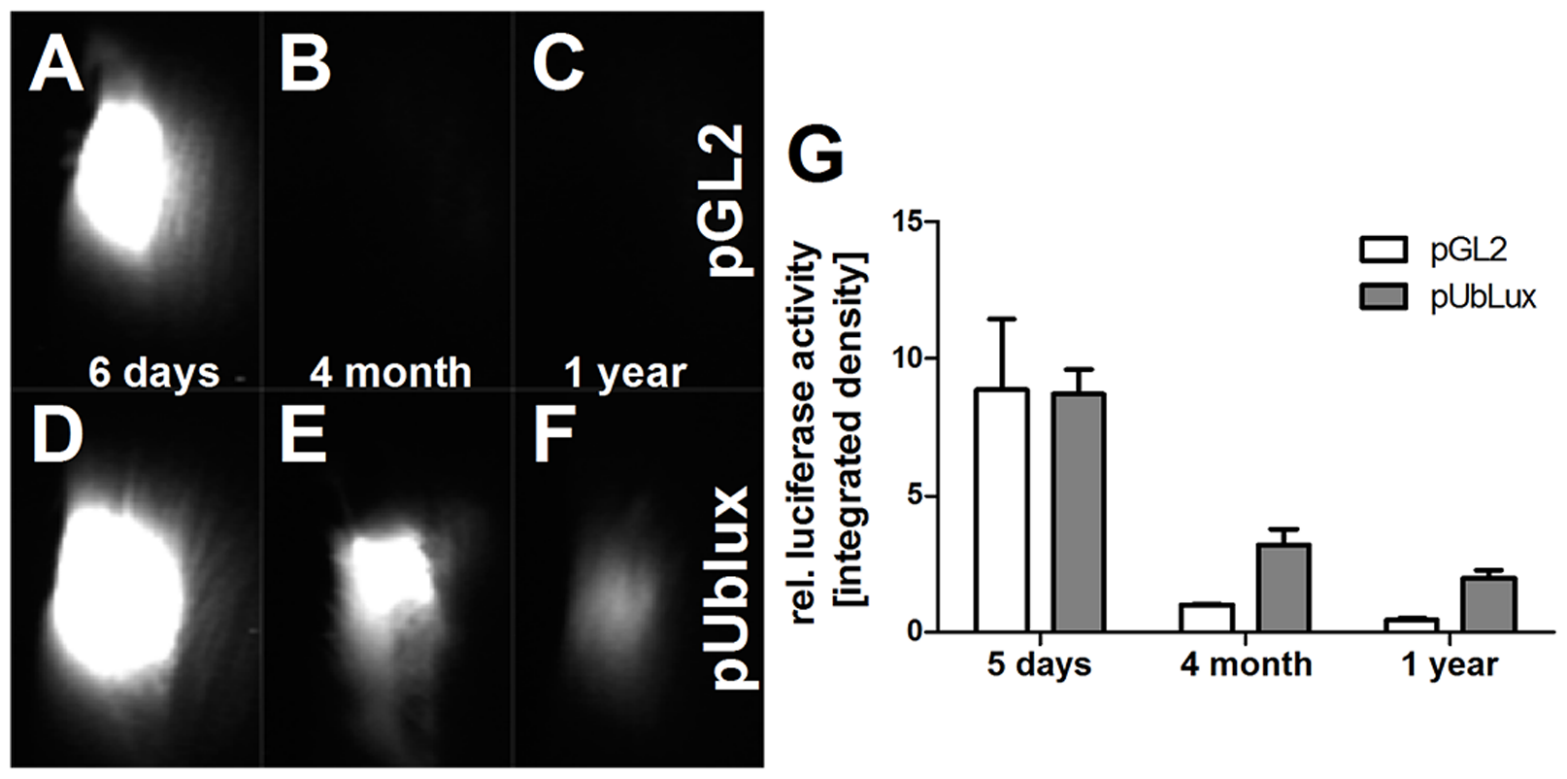
Establishment of long-term therapy using pUblux vector system compared to the regular CMV promoter systems: Rat thigh muscles were transfected with either pGl2 (A–C) or pUblux (D–F) overexpression vector. Luciferase activity was measured in vivo 5 days (A, D), 4 month (B, E) and 1 year (C, F) after transfection and evaluated using ImageJ-software (G).

In CAN, fibrosis develops over a long period of time requiring a long-term gene therapy system. Therefore, we first compared the expression profile of a vector with a eukaryotic promotor with a vector using the classic viral cytomegalie virus (CMV)-promotor for systemic overexpression of luciferase in the rat muscle. Seven days after gene therapy, both vectors showed strong luciferase activity in the transfected muscles (Fig. 1A, D, G). In contrast, four months later muscle luciferase activity was nearly absent in rats treated with the CMV-promotor driven plasmid but still showed high activity in the rats treated with the ubiquitin-promotor driven plasmid (Fig. 1B, C, G). This activity was further declined 1 year after gene therapy but was also still detectable by monitoring muscle luciferase activity in vivo (Fig. 1C, F, G). Consequently, in this study TSP-2 gene therapy was done using the ubiquitin-driven vector.

### TSP-1 and TSP-2 were expressed in kidney grafts in the F344/Lew model

**Figure 2:**
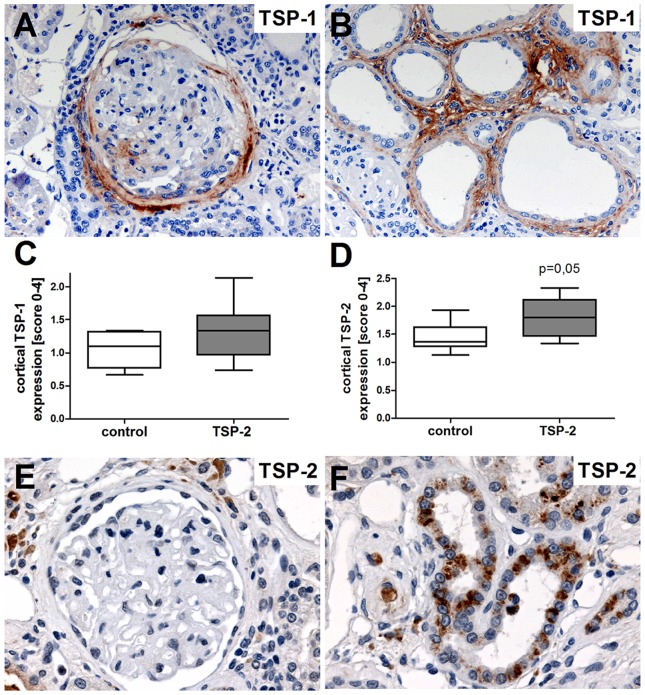
Expression of TSP-1 and TSP-2 in F344 Lewis rat renal allograft model. Renal TSP-1 and TSP-1 expression was evaluated in biopsies from rats treated with the overexpression plasmid for luciferase control or TSP-2 using immunohistochemistry. TSP-1 expression was localized in glomeruli and within and around the Bowman's capsule (A, brown staining) as well as in fibroblasts and atrophic tubular cells (B). A semiquantitative evaluation of TSP-1 expression revealed no significant differences between the two groups (C). Staining for endogenous TSP-2 showed marked expression within some tubules (F), while glomerular staining for TSP-2 is lacking (E). Endogenous TSP-2 expression shows a tendency to increased TSP-2 expression within the TSP-2 treated group compared to the controls. Control (n = 8) vs. TSP-2 treated (n = 8).

In healthy rat kidneys, TSP-1 as well as TSP-2 staining was absent within the glomeruli and rarely found within the tubulointerstitial compartment (data not shown, see [15]). In contrast, thirty weeks after kidney transplantation remarkable TSP-1 staining could be detected within the Bowman's capsule and to a lesser extent in the glomerular tuft of glomeruli with pronounced pathological changes (Fig. 2A). In addition, within the tubulointerstitial compartment atrophic tubules and in particular interstitial fibroblasts also express high levels of TSP-1 (Fig. 2B) indicating an important role of TSP-1 in disease progression of CAN. However, TSP-1 expression was comparable in both investigated groups (Fig. 2C). Furthermore, endogenous TSP-2 was rarely expressed in the glomeruli from transplanted grafts (Fig. 2E) and within the tubulointerstitial compartment (Fig. 2F). Semiquantitative evaluation of TSP-2 staining patterns did show a tendency to slightly higher TSP-2 expression in the TSP-2 gene therapy group compared to the control (Fig. 2D).

### Renal injury was impaired by TSP-2 gene therapy in the CAN model

**Figure 3:**
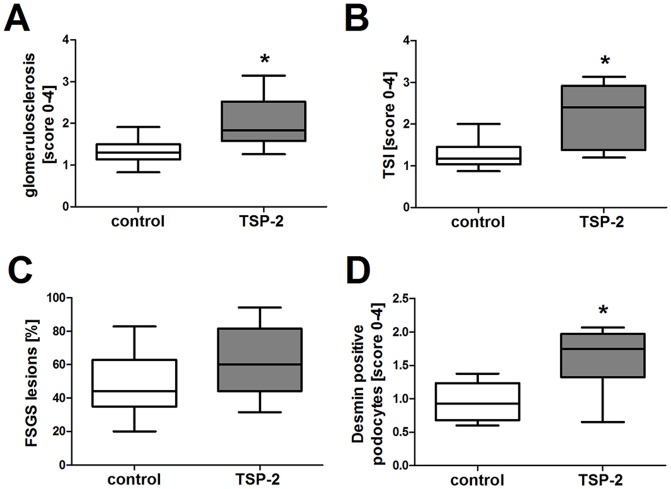
Renal injury was impaired by TSP-2 gene therapy. Injury in renal transplants was assessed by semiquantitative scoring of glomerulosclerosis (A) and tubular injury (B) in PAS-stained paraffin sections as well as FSGS-lesions (C). Podocyte damage was evaluated by semiquantitative scoring of desmin positive podocytes by immunohistochemistry (D). Control (n = 8) vs. TSP-2 treated (n = 8); *p<0,02.

**Figure 4:**
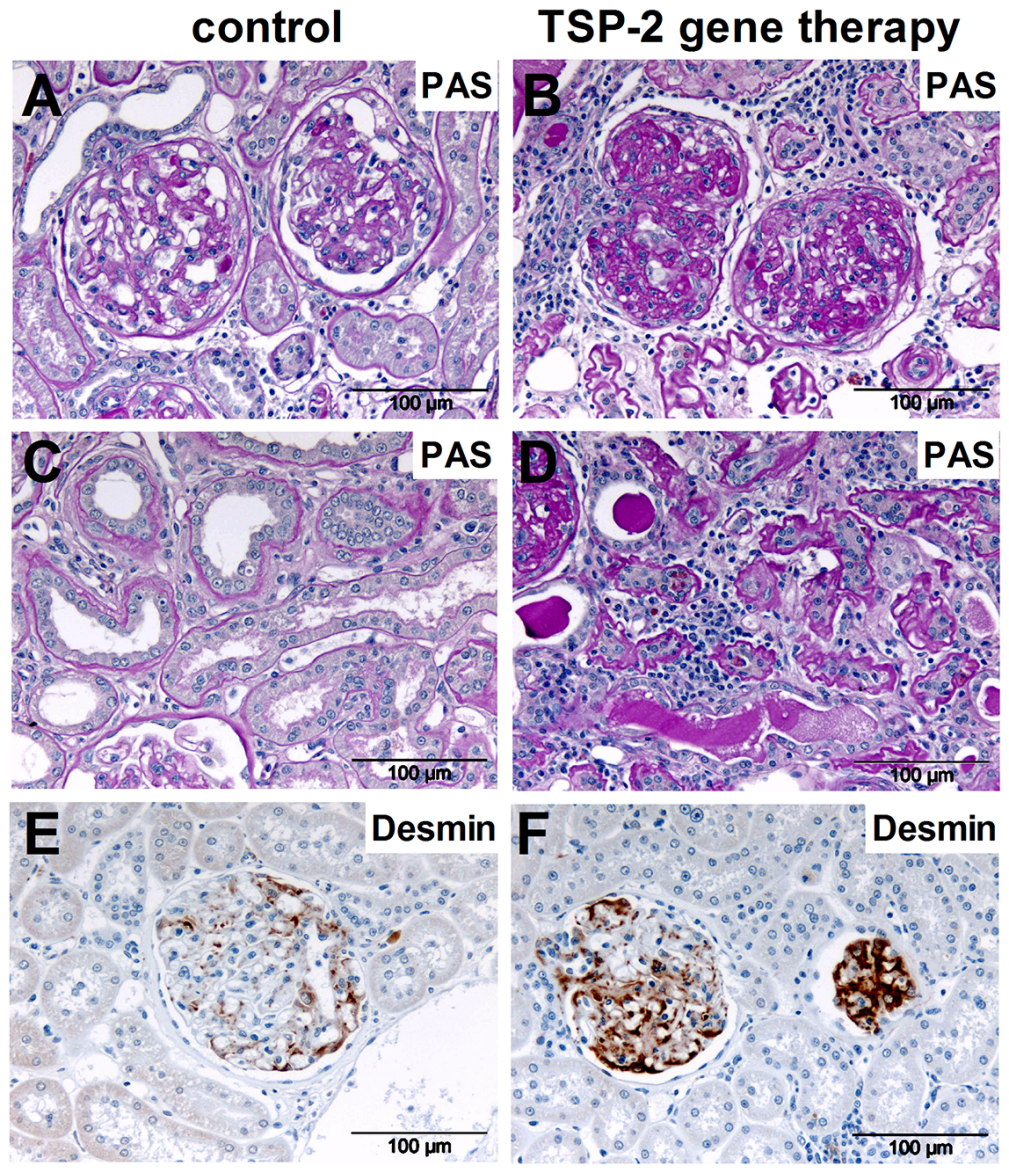
PAS and desmin staining in renal grafts treated with TSP-2 gene therapy. Representative microphotographs from PAS staining of kidney grafts showing glomerular (A, control plasmid; B, TSP-2 therapy) and tubulointerstitial changes (C, control plasmid; D, TSP-2 therapy) are shown. Glomerular desmin staining using immunohistochemistry is shown as a marker of injured podocytes (E, control plasmid; F, TSP-2 therapy, brown staining).

At the endpoint of our study, glomerulosclerotic changes as well as tubulointerstitial injury including cast formation, tubular atrophy and fibrosis could be seen in both the control and the TSP-2 treated group. We expected that TSP-2 gene therapy would ameliorate histomorphological outcome and kidney function from transplanted kidneys by inhibition of TSP-1 mediated TGF-β activation and its anti-inflammatory effects. Surprisingly, glomerulosclerosis (Fig. 3A; 4A–B) and tubulointerstitial injury (Fig. 3B, 4C–D) in the graft were more severe in the group treated with TSP-2 gene therapy compared to the control plasmid treated rats. FSGS-lesions occurred more frequently in the TSP-2 overexpressing group, but this effect did not reach statistical significance (Fig. 3C). Podocyte injury, as assessed by desmin staining, was significantly increased in the rats treated with TSP-2 gene therapy (Fig. 3D). As shown in representative pictures in Fig. 4E-F, desmin staining could be detected more frequently in podocytes and with increased intensity in the TSP-2 treated group.

**Figure 5:**
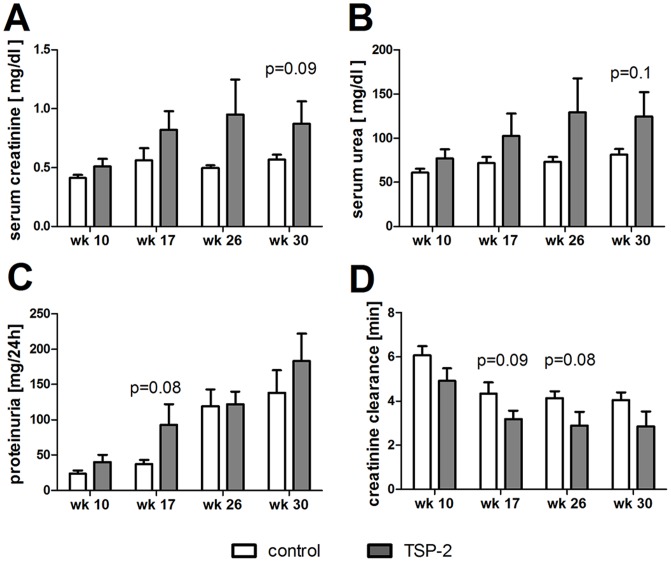
Kidney function after overexpression of TSP-2 in the chronic renal allograft model. Renal function was evaluated 10, 17, 26 and 30 weeks after kidney transplantation in rats treated with either TSP-2 or control gene therapy, as assessed by serum creatinine (A), serum urea (B), proteinuria (C) and creatinine clearance (D).

In addition, several parameters relating to kidney function (serum creatinine or urea, creatinine clearance, proteinuria) uniformally showed a tendency towards impairment via application of TSP-2 gene therapy, although statistical significance was not reached (p-values between 0,08 and 0,10, Fig. 5A–D).

### Overexpression of TSP-2 reduced TGF-β activation in the rat CAN model without effects on matrix accumulation

**Figure 6:**
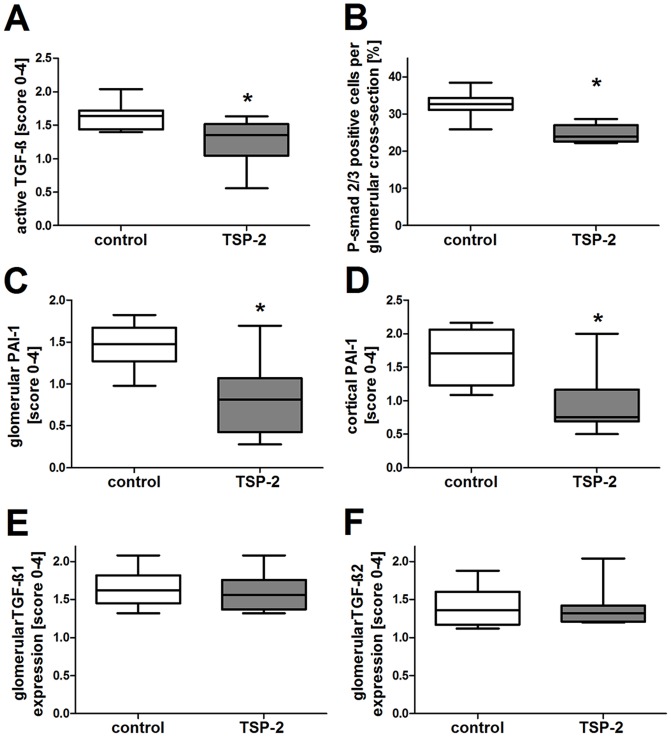
TSP-2 gene therapy reduced TGF-β activation while not affecting total TGF-β expression. In gene therapy, treated rats with renal transplants TGF-β were evaluated by immunohistochemistry. Active TGF-β was either detected directly by using an antibody recognizing active TGF-β (A) or indirectly by evaluation of phosphorylation of the TGF-β signaling molecule smad2/3 (B) or expression of the TGF-β downstream target PAI-1 within the glomeruli (C) or the cortex (D). Representative microphotographs of PAI-1 staining in renal grafts treated with control (E) and TSP-2 overexpressing plasmid (F) are shown. Total TGF-β1 (G) and TGF-β2 (H) was similar in both groups. Control (n = 8) vs. TSP-2 treated (n = 8); *p<0,05.

**Figure 7:**
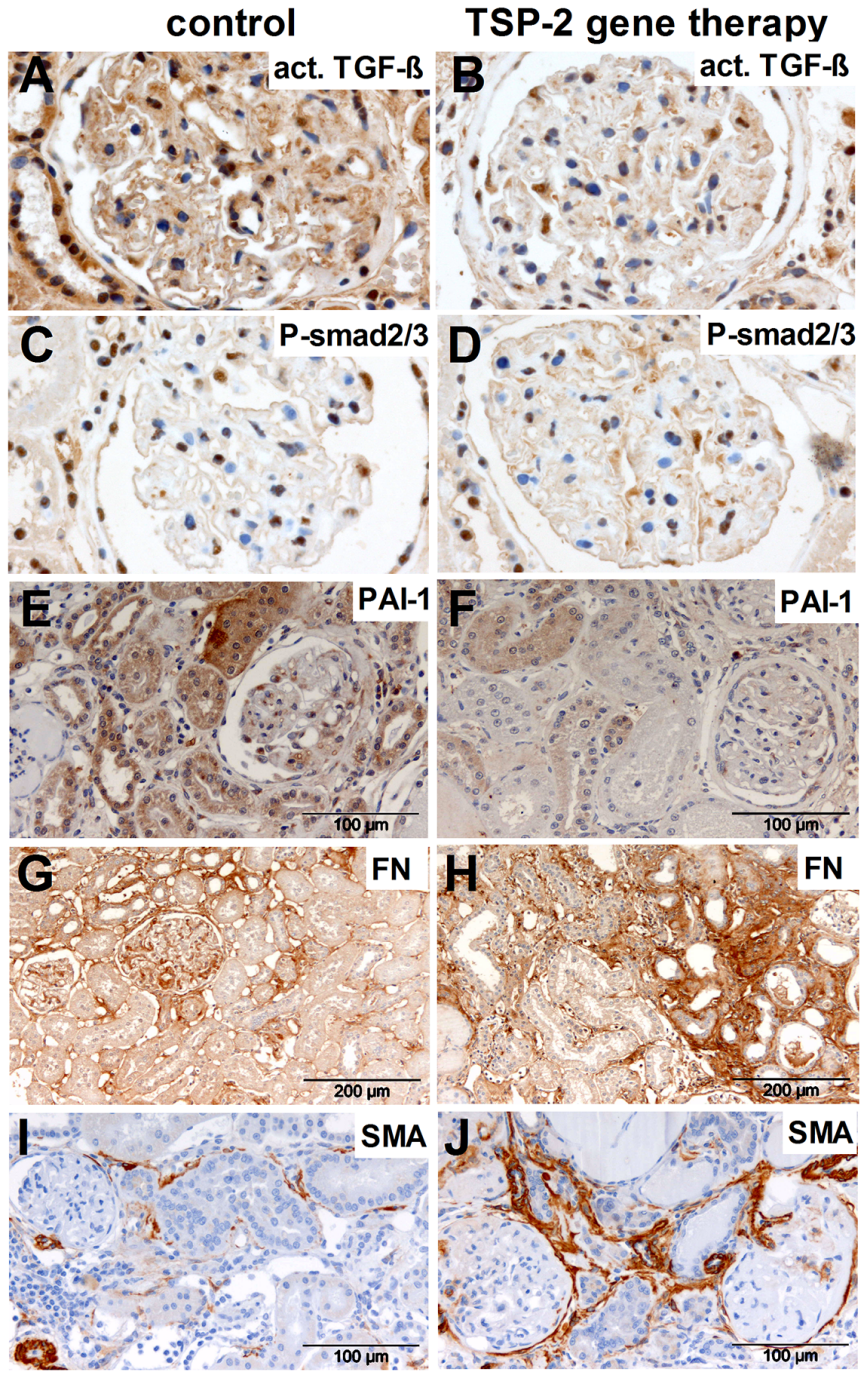
Fibronectin and alpha-smooth muscle actin staining in renal grafts treated with TSP-2 gene therapy. Representative microphotographs from immunohistological staining of kidney grafts for active TGF-β (A, control plasmid; B, TSP-2 therapy, brown cytosolic staining), P-smad 2/3 (C, control plasmid; D, TSP-2 therapy, brown nuclear staining), PAI-1 (E, control plasmid; F, TSP-2 therapy, brown staining), fibronectin (G, control plasmid; H, TSP-2 therapy, brown staining) and alpha-smooth muscle actin (I, control plasmid; J, TSP-2 therapy, brown staining) are shown.

**Figure 8:**
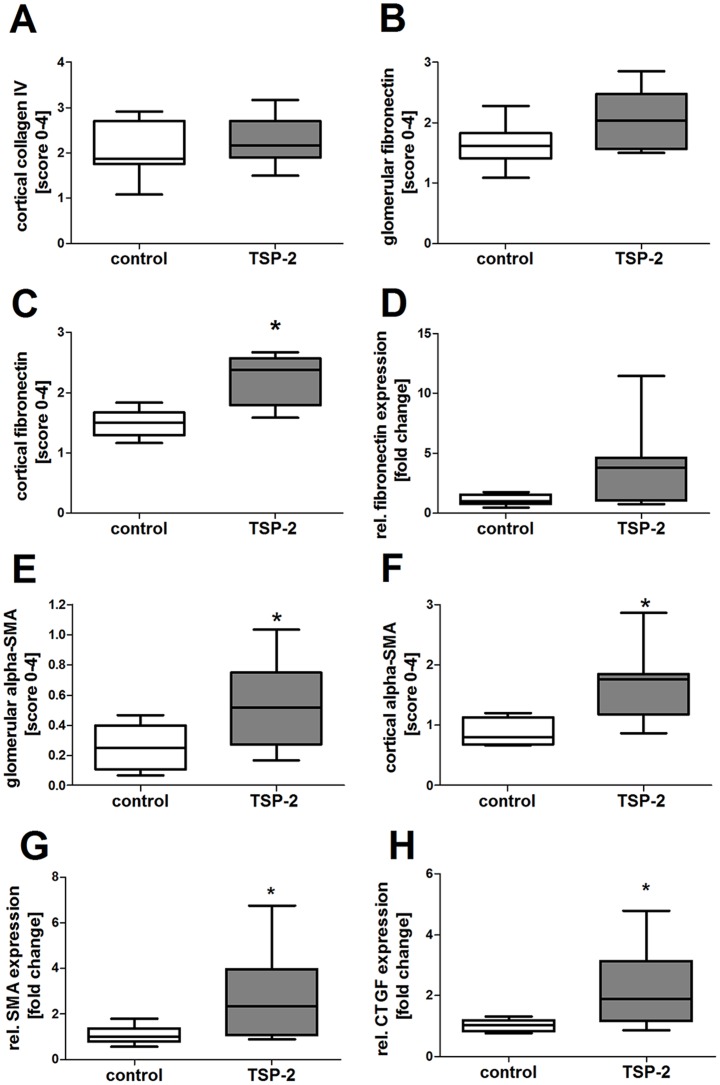
Matrix deposition is not ameliorated by TSP-2 gene therapy in the chronic allograft model. Collagen IV, fibronectin and alpha-smooth muscle actin (SMA) were evaluated by semiquantitative scoring of immunohistologic staining. Collagen IV was similar in cortex (A) of both groups. Fibronectin was similar in glomeruli (B) but increased within the cortex of renal grafts from TSP-2 treated animals (C). Glomerular fibronectin mRNA shows a tendency to higher expression in TSP-2 treated animals (D) Myofibroblasts were evaluated in glomeruli (E) as well as in renal cortex (F) using alpha-smooth muscle actin (SMA) as a marker. Glomerular mRNA of SMA (G) and CTGF (H) was increased in TSP-2 treated rats compared to control. Control (n = 8) vs. TSP-2 treated (A–C, E–F:n = 8; D, G–H:n = 6); *p<0,05.

Next, we evaluated whether TSP-2 gene therapy could inhibit TSP-1 mediated TGF-β activation in the rat CAN model as demonstrated before in the anti-Thy1 model (16). In this study, we confirmed the inhibitory effect of TSP-2 overexpression on TGF-β activation by first, reduced amounts of active TGF-β using a specific antibody (Fig. 6A; 8A,B) and second, via demonstrating a reduced number of glomerular cells positive for the phosporylated form of the TGF-β signal transduction molecule smad 2/3 (Fig. 6B; 8C,D) and third, via demonstrating a significantly reduced expression of the TGF-β downstream target plasmin activator inhibitor -1 (PAI-1) within glomerular (Fig. 6C) as well as cortical areas (Fig. 6D) when the TSP-2 treated rats were compared with the control group. PAI-1 staining could be clearly detected in tubules as well as in podocytes in biopsies from the control group (Fig. 7E) but could only hardly detected in kidneys from TSP-2 gene therapy treated rats (Fig. 7F).

In contrast, total TGF-β as assessed by immunostaining for TGF-β1 (Fig. 6E) and TGF-β 2 (Fig. 6F) were similar in both groups.

Unexpectedly, renal matrix accumulation, as assessed by expression of collagen IV (Fig. 8A) or fibronectin (Fig. 8B–C), was not reduced by treatment with TSP-2 gene therapy compared to the control group. Cortical fibronectin was even significantly higher in the TSP-2 treated group in immunhistological evaluation (Fig. 8C, 7A,B) and showing a tendency to higher fibronectin expression in quantitative PCR (Fig. 8D). In addition, myofibroblasts as assessed by sm-actin staining, were also increased in glomeruli (Fig. 8E) as well as in the renal cortex (Fig.8F) from rats treated with TSP-2 gene therapy compared to controls. In representative microphotographs, sm-actin staining is shown predominantly around the bowman's capsule, in the tubuloinsterstitial compartment but also in the glomeruli in TSP-2 gene therapy treated kidneys (Fig. 7D). In contrast, in renal grafts from control rats only weak sm-actin can be detected (Fig. 7C). Glomerular sm-actin was also significantly upregulated on mRNA level in TSP-2 treated rats (Fig. 7G). In addition, mRNA expression of the pro-fibrotic connective tissue growth factor (CTGF) was also upregulated in glomeruli from TSP-2 treated rats.

### Renal graft inflammation was reduced by TSP-2 gene therapy

**Figure 9:**
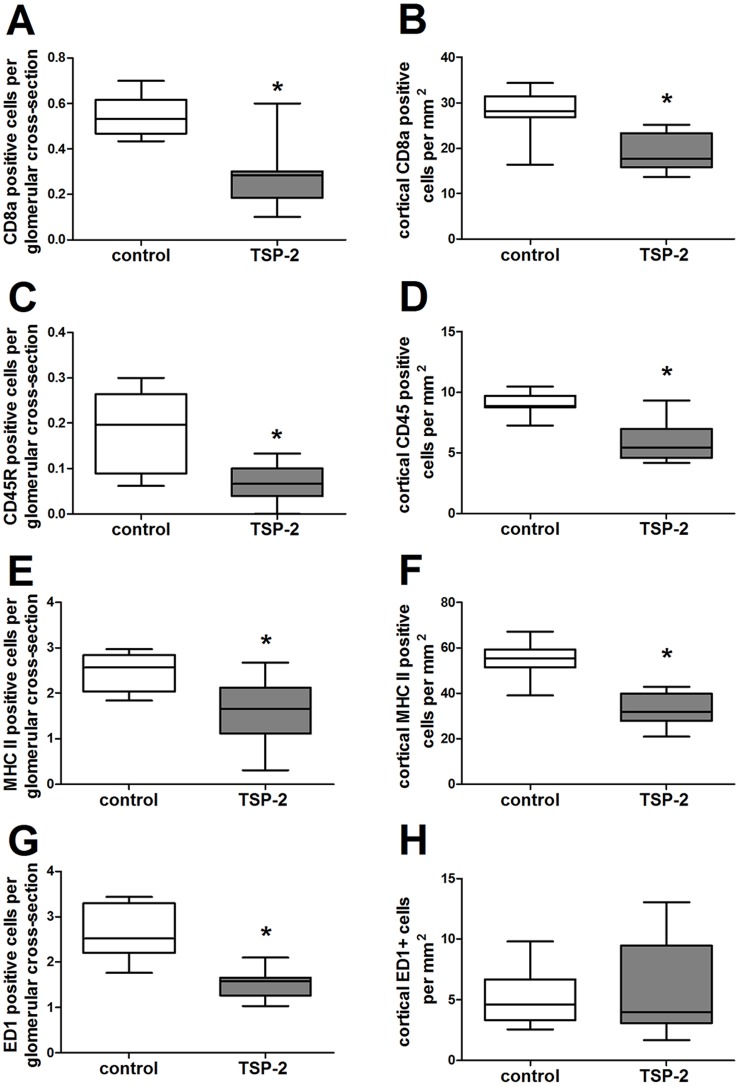
Renal graft inflammation was reduced by TSP-2 gene therapy. Graft inflammation was evaluated by counting CD8a positive T-cells (A, within the glomeruli; B cortical), CD45R positive B-cells (C, glomerular; D, cortical), MHC II positive antigen presenting cells (E, glomerular; F, cortical) and ED-1 positive macrophages (G, glomerular; H, cortical) after staining by immunohistochemistry. Control (n = 8) vs. TSP-2 treated (n = 8); *p<0,02.

**Figure 10:**
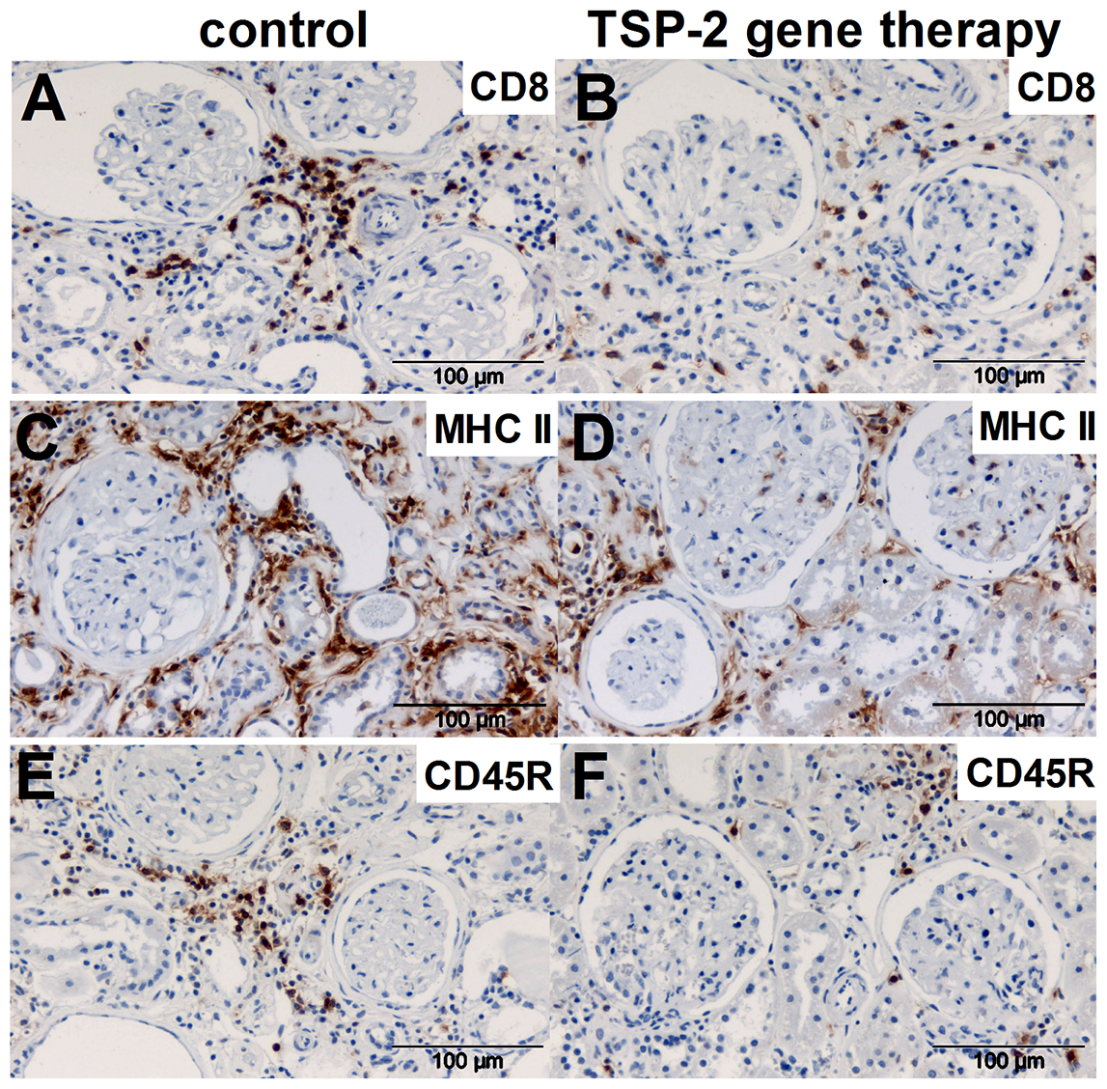
Immunohistochemistry of inflammatory cells in renal grafts after TSP-2 gene therapy. Representative microphotographs from immunohistological staining of kidney grafts for CD8a positive T-cells (A, control plasmid; B, TSP-2 therapy, brown staining), MHC II positive antigen presenting cells (C, control plasmid; D, TSP-2 therapy, brown staining) and CD45R positive B-cells (E, control plasmid; F, TSP-2 therapy, brown staining) are shown.

Inflammatory cells are known mediators of kidney fibrosis. Therefore, we analyzed influx of T-cells, B-cells, macrophages and MHC II –positive inflammatory cells in the grafts. Within the kidney, inflammatory cells were predominantly detected within the tubulointerstitial compartment (Fig. 8–9). Nevertheless, within glomeruli CD8a-positive T-cells (Fig. 9A), CD45R-positive B-cells (Fig. 9C), MHC II-positive inflammatory cells (Fig. 9E) and ED-1 positive macrophages (Fig. 9G) were significantly lower in rats treated with the TSP-2 overexpression plasmid compared to the animals treated with the control vector. Similarly, within the renal cortex CD8a-positive T-cells (Fig. 9B), CD45R-positive B-cells (Fig. 9D) and MHC II-positive inflammatory cells (Fig. 9F) were significantly lower in TSP-2 treated grafts compared to the control group. Only ED1-positive macrophages were found in comparable numbers in the cortex, but this cell population shows the lowest abundance from all inflammatory cells investigated (Fig. 9H). Representative microphotographs shows (Fig. 10A–F) graft inflammation predominantly located in the tubulointerstitial compartment.

### TSP-2 gene therapy reduced capillary density in the kidney graft by reducing endothelial cell proliferation

During CAN, microvascular rarefaction is a frequently reported phenomenon influencing the graft function negatively. Considering our unexpected divergent results regarding an increase of glomerulosclerosis and IF/TA despite inhibition of TGF-β activation and inflammation via TSP-2 gene therapy in Fischer-Lewis CAN, we considered the antiangiogenic long-term properties of TSP-2 as a possible explanation and therefore investigated capillary density by immunohistological staining for CD31.

**Figure 11:**
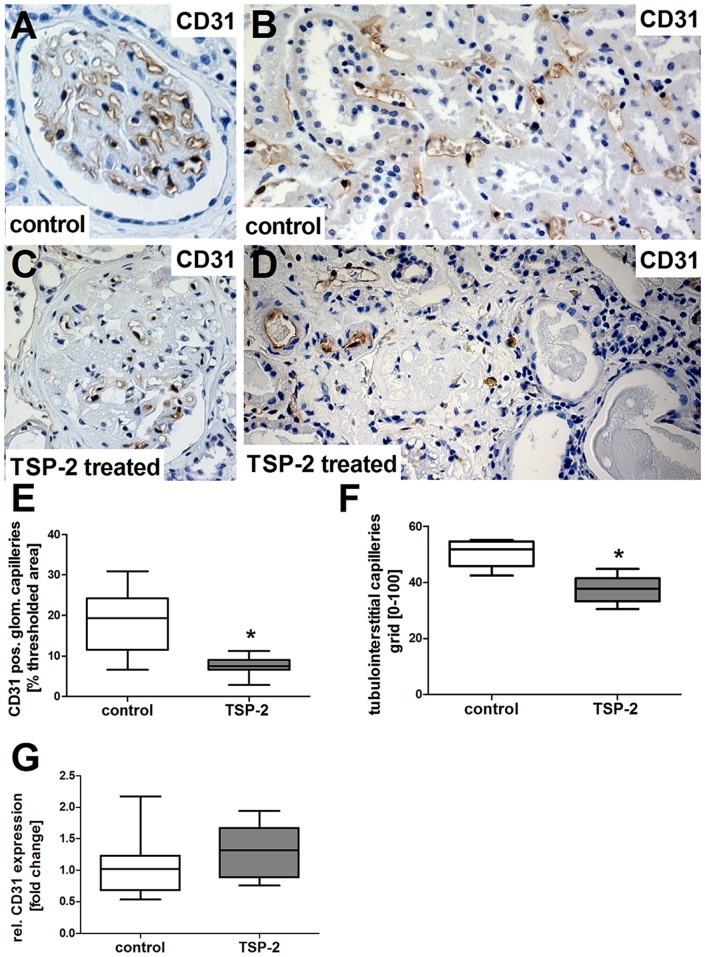
Capillary rarefaction in renal grafts treated with TSP-2 gene therapy. Representative pictures of renal grafts after immunohistologic staining for CD31 within the glomeruli (A) and tubulointerstitial compartment (B) of kidney-transplanted rats treated with the control plasmid. Renal grafts from rats treated with TSP-2 showed considerable rarefaction of capillaries in the glomeruli (C) and the tubulointerstitium (D). Capillary rarefaction was analyzed by computer-assisted image analysis in the glomeruli (E) or using a grid in the tubulointerstitium (F). CD31 mRNA was analyzed in glomerular extracts in grafts from control and TSP-2 treated rats using real-time PCR. Control (n = 8) vs. TSP-2 treated (E-F:n = 8; G:n = 6); *p<0,008.

**Figure 12:**
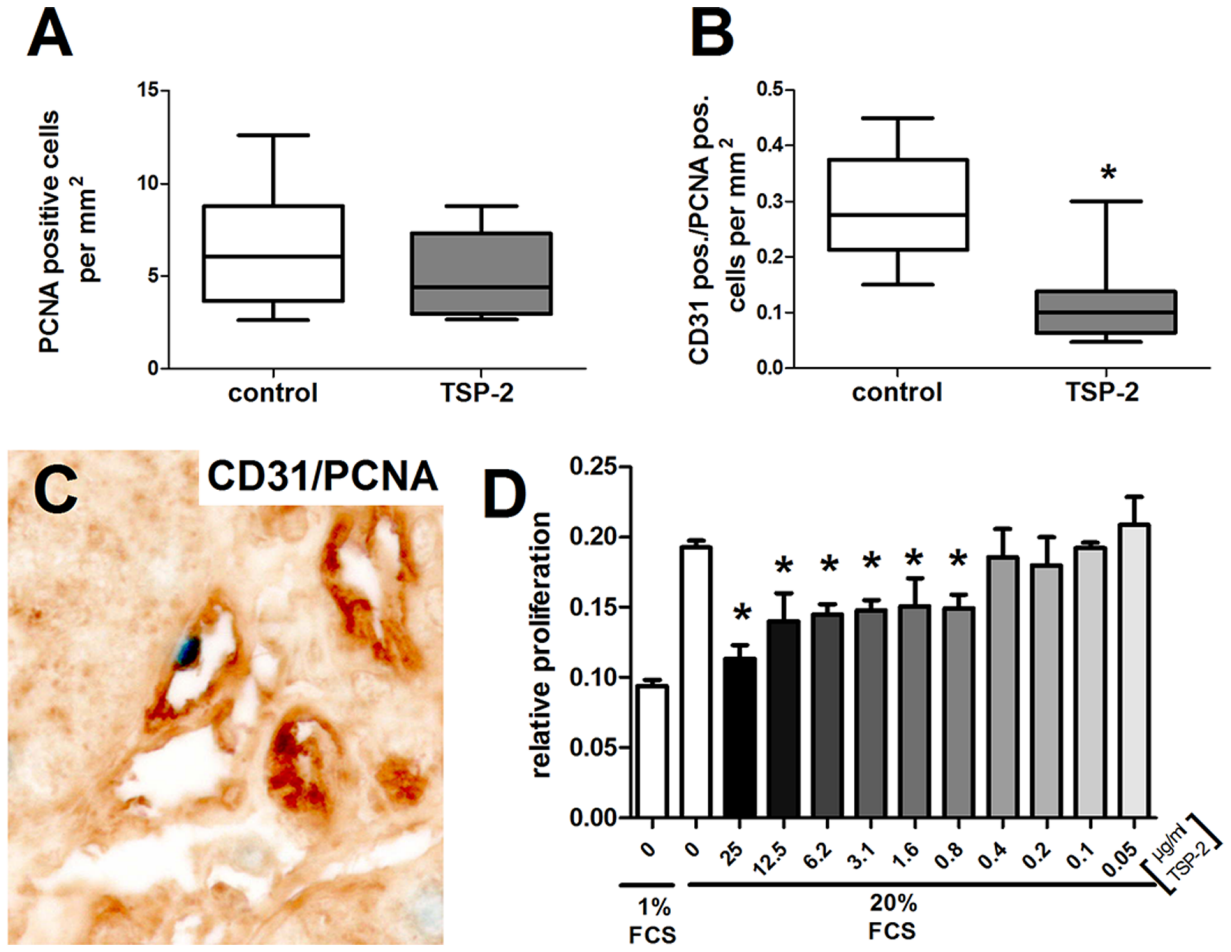
TSP-2 inhibits renal endothelial cell proliferation. Since total renal proliferation, as assessed by PCNA staining, was similar in both groups (A), TSP-2 gene therapy specifically reduced endothelial proliferative activity (B), as assessed by CD31/PCNA double positive cells (C, endothelial cells stained in brown and PCNA positive nuclei in blue). Control (n = 8) vs. TSP-2 treated (n = 8); *p<0,007. The effect of TSP-2 on proliferation of isolated rat endothelial cells was investigated by a BrdU-incorporation assay (D, n = 6; *p<0,05).

Kidney grafts from rats treated with the luciferase control plasmid showed only minor rarefaction of glomerular (Fig. 11A, brown staining) as well as peritubular capillaries (Fig. 11B, brown staining) compared to healthy kidneys in earlier studies [29]. In contrast, glomerular and peritubular CD31 staining from TSP-2 treated rats was rarely detected (Fig. 11C, D, brown staining). Computer-assisted quantification of CD31-positive staining confirmed that TSP-2 gene therapy significantly reduced glomerular staining (Fig. 11E). However, glomerular CD31 mRNA expression was comparable in both groups (Fig. 11G). In addition, peritubular capillaries, as assessed by mean CD31 positive fields of a 10×10 grid, were significantly reduced by 25% in the TSP-2 treated group compared to the control group (Fig. 11F). Increased loss of capillaries is mediated by a reduced proliferative activity specifically of endothelial cells as indicated by double staining for CD31 and PCNA. Total renal proliferation, as assessed by the proliferation marker PCNA, was low and not significantly different in grafts of both groups at the endpoint of our study (Fig. 12A). However, the number of CD31/PCNA double positive cells (Fig. 12C, PCNA nuclear blue staining and CD31 brown staining) was about 60% lower in TSP-2 treated rats compared to controls (Fig. 12B).

**Figure 13:**
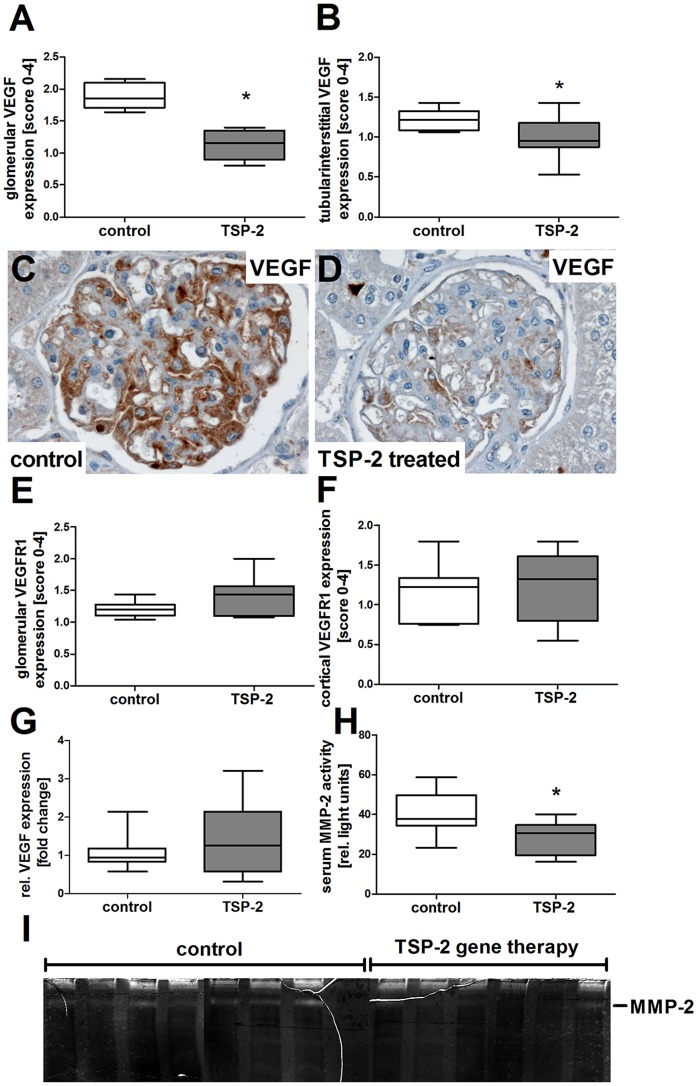
Influence of TSP-2 gene therapy on VEGF/VEGF receptor expression and MMP-2 activity. VEGF and VEGF receptor 1 expression was assessed by semiquantitative scoring of immunohistochemistry. VEGF was reduced in glomeruli (A) as well as in the tubulointerstitial compartment (B) from TSP-2 treated rats. Representative pictures from VEGF stained glomeruli showing pronounced VEGF expression in podocyted as well as mesangial cells in kidneys from control plasmid treated animals (C, brown staining). In contrast, VEGF was only rarely detected in glomeruli from TSP-2 treated rats (D, brown staining). Expression of VEGF receptor 1 was similar in glomeruli (E) as well as cortex (F) of both groups. Glomerular VEGF mRNA expression was comparable in both groups (G). Serum MMP-2 activity at endpoint of the study was detected by zymography (I) and evaluated by densitometry (H). Control (n = 8) vs. TSP-2 treated (A–F:n = 8; G–I:n = 6); *p<0,05.

Suggesting a direct antiproliferative effect of TSP-2 for renal endothelial cells, incubation of isolated rat glomerular endothelial cells *in vitro* resulted in significant reduction of the proliferative response (Fig. 12D). Inhibition of endothelial cell proliferation started at concentrations of 0.8 µg/ml TSP-2 and reached a peak inhibitory effect of 41.3±5.2% when a concentration of 25 µg/ml was used (Fig. 12D). In our rat CAN model, TSP-2 mediated inhibition of endothelial cell proliferation was linked to a reduction of the VEGF. In control plasmid treated rats we could detect strong VEGF-expression within podocytes and to a lower extent also within the mesangium (Fig. 13C). In contrast, glomeruli from TSP-2 (Fig. 13A, B, D) compared to control vector (Fig.13A, B, C) treated rats expressed VEGF at a significantly lower level as indicated by semi-quantitative evaluation of glomerular as well as tubulointerstitial VEGF. However, glomerular VEGF mRNA expression was similar in both groups at the end of the experiment (Fig. 13G), suggesting regulation of VEGF at the posttranslational level. Glomerular as well as tubulointerstitial expression of the VEGF receptor 1 was similar in both groups (Fig. 13E, F). Since TSP-2 is a known regulator of MMP-2 we analysed serum samples for MMP-2 activity using zymography. Interestingly, MMP-2 activity was slightly lower in sera from rats treated with the TSP-2 plasmid compared to samples derived from control plasmid treated rats (Fig. 13H,I).

## Discussion

Kidney transplantation is the best renal replacement therapy, but graft survival is frequently limited by chronic allograft injury which is characterized by progressive interstitial fibrosis, tubular atrophy (IF/TA) as well as well as microvascular damage and fibrosis [30]. TGF-β as the key mediator for kidney fibrosis is also a target for anti-fibrotic strategies in man but may also be accompanied by side effects considering its overall importance in many cellular processes including tumor growth [6]. Our group identified TSP-1 as the major endogenous activator of TGF-β in different experimental renal diseases like mesangial proliferative glomerulonephritis [23], [25] as well as in diabetic nephropathy [31]. The link of TSP-1 and TGF-β activation in several experimental kidney disease models [22], [23], [25], [32]-[34] as well as the fact of *de novo* upregulation of TSP-1 as an early response gene at sites of injury during experimental and human kidney disease [35], [36] suggests TSP-1 as a better and more specific anti-fibrotic target for kidney disease compared to overall blockade of TGF-β, since TGF-β activation not mediated via TSP-1 will not be affected.

Therefore, in this study we aimed for the blocking of TSP-1 mediated TGF-β activation and for the reduction of renal inflammation in the well-established Fischer-Lewis rat model of CAN 28 weeks after the start of the long-term TSP-2 gene therapy. TSP-1 has been detected in our model in injured areas supporting the profibrotic role of TSP-1 also according to human kidney transplantation studies [35], [37].

For the first time, we demonstrate that a one-time gene transfer technigue is able to work for almost 7 months in a rat model of CAN. In accordance to our previous short term (one week) study in an acute glomerulonephritis model [15], we now demonstrate that the apparent competition of long-term TSP-2 therapy with endogenously increased TSP-1 was able to specifically inhibit TGF-β activation in CAN as assessed at the end point 7 months after application. Furthermore, we could demonstrate marked anti-inflammatory properties during CAN decreasing renal accumulation of T-cells, B-cells and macrophages by long-term TSP-2 gene therapy. Anti-inflammatory properties of endogenous or exogenous TSP-2 were also shown in anti-GBM nephritis [38], anti-Thy1 nephritis [15] as well as in rheumatoid arthritis [39] and myocarditis [16].

TSP-2 seems to exert its anti-inflammatory action by different mechanisms. First, TSP-2 regulates pro-inflammatory mediators. In rheumatoid arthritis, TSP-2 suppressed the production of interferon-gamma and tumor necrosis factor-alpha, and induced the depletion of tissue-residing T-cells [39]. During myocarditis, anti-inflammatory IL-10 expression was significantly lower in TSP-2 deficient mice resulting in reduced numbers of regulatory T-cells [16]. Second, high amounts of either TSP-1 or TSP-2 induce T-cell apoptosis via the CD47 receptor [40].

TGF-β activation and renal inflammation are both known contributors to the development of IF/TA and glomerulosclerosis in the graft and usually are concordantly regulated as well as associated with each other in CAN [6]. Therefore, we expected beneficial effects of TSP-2 therapy via multiple pathways during experimental CAN in our study. In contrast, matrix accumulation, as monitored by collagen and fibronectin expression, was not inhibited by TSP-2 gene therapy in our CAN model. In addition, myofibroblast conversion a hallmark during kidney disease progression frequently shown to be mediated via TGF-β was also significantly increased in the TSP-2 treatment group. Using real-time PCR, we identified CTGF as a potential mediator of myofibroblast conversion significantly upregulated in the TSP-2 treated group. While CTGF expression and action is frequently linked to TGF-β [41], [42], it can also be induced by TGF-β independent pathways, as reported for IL-13 induced CTGF stimulation [43]. Therefore, our data demonstrate a dissociation of the TGF-β/CTGF axis also in our model system, which can explain the unexpected increase in matrix accumulation and myofibroblast conversion despite ameliorated TGF-β activation. The increased matrix accumulation was associated with significantly increased glomerulosclerosis, podocyte injury as well as tubulointerstitial injury and a tendency to impaired graft function in TSP-2 treated rats compared to controls. Microarray analysis using biopsies from human renal allografts showed that increased interstitial fibrosis is linked to TSP-2 upregulation in kidney grafts [44]. However, the role of endogenous TSP-2 in renal allografts remains unclear. TSP-2 exerted anti-fibrotic effects in experimental acute renal disease [15], [38], myocarditis [16] and age-related cardiomyopathy [17], but displayed profibrotic properties in other diseases like scleroderma [45] or in cardiac cell grafts [46].

Our data suggest that this unusual dissociation of TGF-β activation/inflammation and myofibroblast activation/fibrosis/sclerosis may relate to chronic and long-term anti-angiogenic side effects of the TSP-2 therapy on the renal microvasculature during disease course, which has not been seen during short-term TSP-2 gene therapy in anti-Thy1 nephritis [15]. A recent study emphasized that myofibroblasts originate from detached pericytes resulting in increased fibrosis and capillary loss [47] as found in our study. Whether TSP-2 overexpression causes directly (or indirectly via its anti-angiogenic effects) pericyte detachment is still unclear.

Kidney transplantation followed by long-term TSP-2 gene therapy was associated with a considerable decrease in glomerular as well as peritubular capillary density in our model and could be shown to be due to decreased endothelial cell proliferation *in vivo* as well as *in vitro*. Studies also suggest that this side effect may be highly relevant for the development and progression of CAN after renal transplantation in man, since this procedure is associated with early loss of peritubular capillaries and the degree of peritubular capillary loss in 3 months protocol biopsies predicts decreased renal function at 1 year [48]. Endogenous anti-angiogenic effects of TSP-2 were described previously in anti-GBM nephritis [38], skin wounds, sponge granulomas, and foreign body reaction [49]-[51] as well as cancer [52]. Inhibition of angiogenesis by TSP-2 is complex and includes binding of TSP-2 to its receptors with induction of either endothelial cell apoptosis [53] or inhibition of cell division [54], [55]. In addition, TSPs can indirectly inhibit angiogenesis by binding and clearance of growth factors like VEGF [56] or by clearing of matrix metalloproteases (MMPs) that are involved in VEGF release from matrix stores via the low density lipoprotein receptor-related protein [57]. In this study, we observed significantly reduced VEGF staining in graft biopsies, but it is unclear if this is directly due to the TSP-2 therapy or indirectly caused by increased podocyte damage. Detection of MMP-2 in serum samples in this study showed reduced activities in rats treated with TSP-2 overexpressing plasmid compared to controls. Therefore, disturbed angiogenesis in the TSP-2 treated groups is potentially also due to reduced VEGF release from matrix stores.

Taken together, our data suggest that long term anti-angiogenic effects of TSP-2 gene therapy causing capillary rarefaction with relative ischemia and hypoxia that drives a profibrotic reaction during CAN, which may be mediated via a TGF-β independent increase of CTGF. Several in vitro and in vivo studies demonstrated that CTGF is stimulated in different kidney cells under hypoxic conditions supporting this possible link [58].

Our study has some limitations. First, in this proof of principle study we monitored kidney function at multiple time points during the experiment but histomorphological information and gene expression analysis were restricted until the endpoint of the study. Second, the F344 Lewis transplantation model cannot be directly compared with human CAN, but mimics many features of human renal disease such as TGF-β activation, matrix accumulation, myofibroblast conversion, and inflammation. Third, despite that we could describe clear biological effects in treated versus control treated rats, we do not know the plasma levels of TSP-2 in this study due to the lack of appropriate anti-mouse TSP-2 ELISAs.

In conclusion, long term TSP-2 gene therapy did not ameliorate but unexpectedly rather worsened the progression of CAN in the Fischer-Lewis rat model despite the *in vivo* proof of TSP-2 and its ability to block both TSP-1 mediated TGF-β activation and cellular inflammation. In this experimental CAN model, long-term TSP-2 gene therapy resulted in worsened renal capillary rarefaction via direct or potentially indirect inhibition of endothelial cell proliferation. These results point to the central importance of capillary vessel repair for long-term outcome of CAN and suggest that for a successful, long-term therapy of TSP-2 or its fragments, its anti-angiogenic properties need to be separated from its anti-TGF-β activating and anti-inflammatory effects.

## References

[1] Lamb KE, Lodhi S, Meier-Kriesche HU (2010) Long-term renal allograft survival in the United States: a critical reappraisal. Am J Transplant 11: 450–462. 20973913 10.1111/j.1600-6143.2010.03283.x

[2] Meier-Kriesche HU, Schold JD, Srinivas TR, Kaplan B (2004) Lack of improvement in renal allograft survival despite a marked decrease in acute rejection rates over the most recent era. Am J Transplant 4: 378–383. 14961990 10.1111/j.1600-6143.2004.00332.x

[3] Pascual M, Theruvath T, Kawai T, Tolkoff-Rubin N, Cosimi AB (2002) Strategies to improve long-term outcomes after renal transplantation. N Engl J Med 346: 580–590. 11856798 10.1056/NEJMra011295

[4] El-Zoghby ZM, Stegall MD, Lager DJ, Kremers WK, Amer H, et al (2009) Identifying specific causes of kidney allograft loss. Am J Transplant 9: 527–535. 19191769 10.1111/j.1600-6143.2008.02519.x

[5] Campistol JM, Inigo P, Larios S, Bescos M, Oppenheimer F (2001) Role of transforming growth factor-beta1 in the progression of chronic allograft nephropathy. Nephrol Dial Transplant 16 Suppl 1114–116. 10.1093/ndt/16.suppl_1.114 11369837

[6] Djamali A, Samaniego M (2009) Fibrogenesis in kidney transplantation: potential targets for prevention and therapy. Transplantation 88: 1149–1156. 19935366 10.1097/TP.0b013e3181bcccea PMC2784657

[7] Harris S, Coupes BM, Roberts SA, Roberts IS, Short CD, et al (2007) TGF-beta1 in chronic allograft nephropathy following renal transplantation. J Nephrol 20: 177–185. 17514622

[8] Einecke G, Reeve J, Sis B, Mengel M, Hidalgo L, et al (2010) A molecular classifier for predicting future graft loss in late kidney transplant biopsies. J Clin Invest 120: 1862–1872. 20501945 10.1172/JCI41789 PMC2877953

[9] Yin ZK, Wu XH, Xia YG, Luo CL (2011) Transforming growth factor-beta1 short hairpin RNA inhibits renal allograft fibrosis. Chin Med J (Engl) 124: 655–663. 21518553

[10] Lawrence DA (1996) Transforming growth factor-beta: a general review. Eur Cytokine Netw 7: 363–374. 8954178

[11] Hugo C, Daniel C (2009) Thrombospondin in renal disease. Nephron Exp Nephrol 111: e61–66. 19182492 10.1159/000198235

[12] Ribeiro SM, Poczatek M, Schultz-Cherry S, Villain M, Murphy-Ullrich JE (1999) The activation sequence of thrombospondin-1 interacts with the latency-associated peptide to regulate activation of latent transforming growth factor-beta. J Biol Chem 274: 13586–13593. 10224129 10.1074/jbc.274.19.13586

[13] Young GD, Murphy-Ullrich JE (2004) The tryptophan-rich motifs of the thrombospondin type 1 repeats bind VLAL motifs in the latent transforming growth factor-beta complex. J Biol Chem 279: 47633–47642. 15342643 10.1074/jbc.M404918200

[14] Schultz-Cherry S, Chen H, Mosher DF, Misenheimer TM, Krutzsch HC, et al (1995) Regulation of transforming growth factor-beta activation by discrete sequences of thrombospondin 1. J Biol Chem 270: 7304–7310. 7706271 10.1074/jbc.270.13.7304

[15] Daniel C, Wagner A, Hohenstein B, Hugo C (2009) Thrombospondin-2 therapy ameliorates experimental glomerulonephritis via inhibition of cell proliferation, inflammation, and TGF-beta activation. Am J Physiol Renal Physiol 297: F1299–1309. 19726547 10.1152/ajprenal.00254.2009

[16] Papageorgiou AP, Swinnen M, Vanhoutte D, Vandendriessche T, Chuah M, et al (2012) Thrombospondin-2 prevents cardiac injury and dysfunction in viral myocarditis through the activation of regulatory T-cells. Cardiovasc Res 94: 115–124. 22308237 10.1093/cvr/cvs077

[17] Swinnen M, Vanhoutte D, Van Almen GC, Hamdani N, Schellings MW, et al (2009) Absence of thrombospondin-2 causes age-related dilated cardiomyopathy. Circulation 120: 1585–1597. 19805649 10.1161/CIRCULATIONAHA.109.863266

[18] Gill DR, Smyth SE, Goddard CA, Pringle IA, Higgins CF, et al (2001) Increased persistence of lung gene expression using plasmids containing the ubiquitin C or elongation factor 1alpha promoter. Gene Ther 8: 1539–1546. 11704814 10.1038/sj.gt.3301561

[19] Johnson RJ, Pritzl P, Iida H, Alpers CE (1991) Platelet-complement interactions in mesangial proliferative nephritis in the rat. Am J Pathol 138: 313–321. 1704189 PMC1886179

[20] Vogelbacher R, Meister S, Guckel E, Starke C, Wittmann S, et al (2010) Bortezomib and sirolimus inhibit the chronic active antibody-mediated rejection in experimental renal transplantation in the rat. Nephrol Dial Transplant 25: 3764–3773. 20466656 10.1093/ndt/gfq230

[21] Iruela-Arispe L, Gordon K, Hugo C, Duijvestijn AM, Claffey KP, et al (1995) Participation of glomerular endothelial cells in the capillary repair of glomerulonephritis. Am J Pathol 147: 1715–1727. 7495296 PMC1869935

[22] Kang DH, Joly AH, Oh SW, Hugo C, Kerjaschki D, et al (2001) Impaired angiogenesis in the remnant kidney model: I. Potential role of vascular endothelial growth factor and thrombospondin-1. J Am Soc Nephrol 12: 1434–1447. 11423572 10.1681/ASN.V1271434

[23] Daniel C, Takabatake Y, Mizui M, Isaka Y, Kawashi H, et al (2003) Antisense oligonucleotides against thrombospondin-1 inhibit activation of tgf-beta in fibrotic renal disease in the rat in vivo. Am J Pathol 163: 1185–1192. 12937160 10.1016/s0002-9440(10)63478-5 PMC1868256

[24] Keller K, Daniel C, Schocklmann H, Endlich KH, Kerjaschki D, et al (2006) Everolimus inhibits glomerular endothelial cell proliferation and VEGF, but not long-term recovery in experimental thrombotic microangiopathy. Nephrol Dial Transplant 21: 2724–2735. 16861242 10.1093/ndt/gfl340

[25] Daniel C, Wiede J, Krutzsch HC, Ribeiro SM, Roberts DD, et al (2004) Thrombospondin-1 is a major activator of TGF-beta in fibrotic renal disease in the rat in vivo. Kidney Int 65: 459–468. 14717916 10.1111/j.1523-1755.2004.00395.x

[26] Hugo C, Pichler R, Meek R, Gordon K, Kyriakides T, et al (1995) Thrombospondin 1 is expressed by proliferating mesangial cells and is up-regulated by PDGF and bFGF in vivo. Kidney Int 48: 1846–1856. 8587244 10.1038/ki.1995.483

[27] Dimmler A, Haas CS, Cho S, Hattler M, Forster C, et al (2003) Laser capture microdissection and real-time PCR for analysis of glomerular endothelin-1 gene expression in mesangiolysis of rat anti-Thy 1.1 and murine Habu Snake Venom glomerulonephritis. Diagn Mol Pathol 12: 108–117. 12766616 10.1097/00019606-200306000-00007

[28] Laulajainen T, Julkunen I, Haltia A, Knuutila S, Miettinen A, et al (1993) Establishment and characterization of a rat glomerular endothelial cell line. Lab Invest 69: 183–192. 8394476

[29] Wittmann S, Daniel C, Braun A, Vogelbacher R, Shimizu F, et al (2008) The mTOR inhibitor everolimus attenuates the time course of chronic anti-Thy1 nephritis in the rat. Nephron Exp Nephrol 108: e45–56. 18270495 10.1159/000116112

[30] Jevnikar AM, Mannon RB (2008) Late kidney allograft loss: what we know about it, and what we can do about it. Clin J Am Soc Nephrol 3 Suppl 2S56–67. 18309004 10.2215/CJN.03040707 PMC3152273

[31] Daniel C, Schaub K, Amann K, Lawler J, Hugo C (2007) Thrombospondin-1 is an endogenous activator of TGF-beta in experimental diabetic nephropathy in vivo. Diabetes 56: 2982–2989. 17878288 10.2337/db07-0551

[32] Hugo C, Kang DH, Johnson RJ (2002) Sustained expression of thrombospondin-1 is associated with the development of glomerular and tubulointerstitial fibrosis in the remnant kidney model. Nephron 90: 460–470. 11961406 10.1159/000054735

[33] Abrass CK (2000) The nature of chronic progressive nephropathy in aging rats. Adv Ren Replace Ther 7: 4–10. 10672913 10.1016/s1073-4449(00)70001-x

[34] Hugo C, Shankland SJ, Pichler RH, Couser WG, Johnson RJ (1998) Thrombospondin 1 precedes and predicts the development of tubulointerstitial fibrosis in glomerular disease in the rat. Kidney Int 53: 302–311. 9461090 10.1046/j.1523-1755.1998.00774.x

[35] Hotchkiss H, Chu TT, Hancock WW, Schroppel B, Kretzler M, et al (2006) Differential expression of profibrotic and growth factors in chronic allograft nephropathy. Transplantation 81: 342–349. 16477218 10.1097/01.tp.0000195773.24217.95

[36] McGregor B, Colon S, Mutin M, Chignier E, Zech P, et al (1994) Thrombospondin in human glomerulopathies. A marker of inflammation and early fibrosis. Am J Pathol 144: 1281–1287. 7515560 PMC1887455

[37] Altun B, Usalan C, Haznedaroglu IC, Arici M, Ulusoy S, et al (1999) Thrombopoietin and thrombospondin in renal allograft recipients. Blood Coagul Fibrinolysis 10: 233–237. 10456613

[38] Daniel C, Amann K, Hohenstein B, Bornstein P, Hugo C (2007) Thrombospondin 2 functions as an endogenous regulator of angiogenesis and inflammation in experimental glomerulonephritis in mice. J Am Soc Nephrol 18: 788–798. 17287428 10.1681/ASN.2006080873

[39] Park YW, Kang YM, Butterfield J, Detmar M, Goronzy JJ, et al (2004) Thrombospondin 2 functions as an endogenous regulator of angiogenesis and inflammation in rheumatoid arthritis. Am J Pathol 165: 2087–2098. 15579451 10.1016/S0002-9440(10)63259-2 PMC1618704

[40] Lamy L, Foussat A, Brown EJ, Bornstein P, Ticchioni M, et al (2007) Interactions between CD47 and thrombospondin reduce inflammation. J Immunol 178: 5930–5939. 17442977 10.4049/jimmunol.178.9.5930

[41] Sarkozi R, Flucher K, Haller VM, Pirklbauer M, Mayer G, et al (2012) Oncostatin M inhibits TGF-beta1-induced CTGF expression via STAT3 in human proximal tubular cells. Biochem Biophys Res Commun 424: 801–806. 22814105 10.1016/j.bbrc.2012.07.042

[42] Wang Q, Usinger W, Nichols B, Gray J, Xu L, et al (2011) Cooperative interaction of CTGF and TGF-beta in animal models of fibrotic disease. Fibrogenesis Tissue Repair 4: 4. 21284856 10.1186/1755-1536-4-4 PMC3042008

[43] Liu Y, Meyer C, Muller A, Herweck F, Li Q, et al (2011) IL-13 induces connective tissue growth factor in rat hepatic stellate cells via TGF-beta-independent Smad signaling. J Immunol 187: 2814–2823. 21804025 10.4049/jimmunol.1003260

[44] Rodder S, Scherer A, Raulf F, Berthier CC, Hertig A, et al (2009) Renal allografts with IF/TA display distinct expression profiles of metzincins and related genes. Am J Transplant 9: 517–526. 19191772 10.1111/j.1600-6143.2008.02512.x

[45] Kajihara I, Jinnin M, Yamane K, Makino T, Honda N, et al (2012) Increased accumulation of extracellular thrombospondin-2 due to low degradation activity stimulates type I collagen expression in scleroderma fibroblasts. Am J Pathol 180: 703–714. 22142808 10.1016/j.ajpath.2011.10.030

[46] Reinecke H, Robey TE, Mignone JL, Muskheli V, Bornstein P, et al (2012) Lack of thrombospondin-2 reduces fibrosis and increases vascularity around cardiac cell grafts. Cardiovasc Pathol 22: 91–95. 22512900 10.1016/j.carpath.2012.03.005 PMC3401337

[47] Campanholle G, Ligresti G, Gharib SA, Duffield JS (2013) Cellular Mechanisms of Tissue Fibrosis. 3. Novel mechanisms of kidney fibrosis. Am J Physiol Cell Physiol 304: C591–603. 23325411 10.1152/ajpcell.00414.2012 PMC3625718

[48] Steegh FM, Gelens MA, Nieman FH, van Hooff JP, Cleutjens JP, et al (2011) Early loss of peritubular capillaries after kidney transplantation. J Am Soc Nephrol 22: 1024–1029. 21566051 10.1681/ASN.2010050531 PMC3374365

[49] Kyriakides TR, Leach KJ, Hoffman AS, Ratner BD, Bornstein P (1999) Mice that lack the angiogenesis inhibitor, thrombospondin 2, mount an altered foreign body reaction characterized by increased vascularity. Proc Natl Acad Sci U S A 96: 4449–4454. 10200282 10.1073/pnas.96.8.4449 PMC16352

[50] Kyriakides TR, Tam JW, Bornstein P (1999) Accelerated wound healing in mice with a disruption of the thrombospondin 2 gene. J Invest Dermatol 113: 782–787. 10571734 10.1046/j.1523-1747.1999.00755.x

[51] Kyriakides TR, Zhu YH, Yang Z, Huynh G, Bornstein P (2001) Altered extracellular matrix remodeling and angiogenesis in sponge granulomas of thrombospondin 2-null mice. Am J Pathol 159: 1255–1262. 11583953 10.1016/S0002-9440(10)62512-6 PMC1850515

[52] Tomii Y, Kamochi J, Yamazaki H, Sawa N, Tokunaga T, et al (2002) Human thrombospondin 2 inhibits proliferation of microvascular endothelial cells. Int J Oncol 20: 339–342. 11788898

[53] Mirochnik Y, Kwiatek A, Volpert OV (2008) Thrombospondin and apoptosis: molecular mechanisms and use for design of complementation treatments. Curr Drug Targets 9: 851–862. 18855619 10.2174/138945008785909347 PMC2853770

[54] Armstrong LC, Bjorkblom B, Hankenson KD, Siadak AW, Stiles CE, et al (2002) Thrombospondin 2 inhibits microvascular endothelial cell proliferation by a caspase-independent mechanism. Mol Biol Cell 13: 1893–1905. 12058057 10.1091/mbc.E01-09-0066 PMC117612

[55] Oganesian A, Armstrong LC, Migliorini MM, Strickland DK, Bornstein P (2008) Thrombospondins use the VLDL receptor and a nonapoptotic pathway to inhibit cell division in microvascular endothelial cells. Mol Biol Cell 19: 563–571. 18032585 10.1091/mbc.E07-07-0649 PMC2230579

[56] Greenaway J, Lawler J, Moorehead R, Bornstein P, Lamarre J, et al (2007) Thrombospondin-1 inhibits VEGF levels in the ovary directly by binding and internalization via the low density lipoprotein receptor-related protein-1 (LRP-1). J Cell Physiol 210: 807–818. 17154366 10.1002/jcp.20904 PMC3412056

[57] Belotti D, Paganoni P, Manenti L, Garofalo A, Marchini S, et al (2003) Matrix metalloproteinases (MMP9 and MMP2) induce the release of vascular endothelial growth factor (VEGF) by ovarian carcinoma cells: implications for ascites formation. Cancer Res 63: 5224–5229. 14500349

[58] Kroening S, Neubauer E, Wullich B, Aten J, Goppelt-Struebe M (2010) Characterization of connective tissue growth factor expression in primary cultures of human tubular epithelial cells: modulation by hypoxia. Am J Physiol Renal Physiol 298: F796–806. 20032117 10.1152/ajprenal.00528.2009

